# Supplementary material for: Are carbon emissions trading and green financial instruments synergistic? -Comprehensive quantitative research based on content analysis
Source: PLoS One. 2024 Mar 7;19(3):e0298601. doi: 10.1371/journal.pone.0298601 (PMC10919601; doi:10.1371/journal.pone.0298601)
Supplement: S1 Data — (ZIP) [file pone.0298601.s001.zip › Data/地级市Z_F工作报告.zip!/┴╔─■/┼╠╜⌡╩╨/└┤╫╘╣1⁄2╓┌║┼í╛┬φ┐╦╩2╛▌═°í┐_2019.docx]

各位代表：

　　现在，我代表盘锦市人民政府向大会报告工作，请予审议，并请市政协委员和其他列席同志提出意见。

　　一、2018年工作回顾

　　刚刚过去的一年，是贯彻落实党的十九大精神和本届政府依法履职的开局之年。在省委、省政府和市委的坚强领导下，我们坚持以习近平新时代中国特色社会主义思想为指导，深入学习贯彻习近平总书记关于东北、辽宁老工业基地振兴的系列重要讲话精神，认真贯彻落实全省“一带五基地”建设和“五大区域发展战略”决策部署，重实干、强执行、抓落实，较好地完成了市八届人大一次会议确定的主要目标任务，盘锦在新时代呈现出新气象新面貌。

　　（一）牢牢把握第一要务，全市经济正步入高质量发展轨道。多措并举确保经济运行在合理区间，预计全市地区生产总值完成1200亿元，增长5.5%，总量全省第五；一般公共预算收入完成135.7亿元，增长14.2%，其中税收收入占比80.3%，总量全省第四；固定资产投资完成457.5亿元，总量保持全省第三；社会消费品零售总额完成417亿元，增长6%；城镇人均可支配收入39030元，增长7%，位居全省第三；农村人均可支配收入17130元，增长7.5%，位居全省第二。

　　国有经济稳固支撑，民营经济活力释放。辽河油田连续33年保持千万吨规模稳产，近四年来实现首次全面盈利；召开加快民营企业发展大会和产融供需对接会，出台含金量高的配套支持政策，新增民营企业1.7万户，民营经济贡献了58.8%的固定资产投资。坚持省市县领导定点联系推进重点项目，备受瞩目的华锦阿美炼化一体化合作项目在沙特签订重要协议、由准备预备阶段进入加速推进阶段，宝来巴塞尔轻烃综合利用项目、中石油液化天然气工程项目进展顺利，浩业化工高分子材料、中蓝电子产业园二期被列入国家东北振兴三年滚动计划项目篮子，全市亿元以上项目新开工71个、续建207个、竣工82个。

　　全面推行“链长制”促进工业主导产业集群化发展，规上工业增加值增长10%。现代服务业快速成长，盘山快递物流园年处理快件7.3亿单，天天好德石化网等3个工业品平台获评全省特色电商平台；召开旅游业发展大会，出台全域旅游实施意见和支持政策，全市接待国内外游客3048万人次，旅游总收入增长15.1%。坚持创新驱动发展，引进建设产业技术研究院5个，培育科技型中小企业140家、高新技术企业40家，科技成果转化落地61项，高新技术产品增加值增长15%。

　　（二）持之以恒深化改革开放，发展动力活力进一步增强。高标准完成市级机构改革、事业单位改革和开发区整治任务，42个工作部门完成集中挂牌、人员转隶、三定方案制定，301家涉改事业单位整合为37家，开发区由9个优化整合为7个，辽河口经济区委托盘山县管理。“放管服”改革取得新进展，“证照分离”和“双随机、一公开”监管全面推开，市县两级审批服务事项70%以上实现一网一门通办，180个高频事项实现“最多跑一次”。驻盘央企“三供一业”资产及管理权分离移交工作全面完成，鼎翔集团厂办大集体改革积极推进，市属国有企业法人户数和管理层级有效压减，与省属国企开展8个合作项目。农垦改革完成4项国家专项试点任务。县镇财政管理体制改革成效显著，33个镇街建立一级国库，镇级财政收入增长58.9%。推进在建项目和闲置土地“两清理”，盘活闲置土地1100亩。

　　一以贯之举全市之力发展辽东湾，统筹做好开发开放、体制机制创新、社会职能划转等工作，新区成为全省推进自贸区协同发展重点园区。盘锦港随着全省港口资源整合迎来发展新机遇，15个5万吨级泊位投入运营，拓宽型10万吨级航道工程即将开工，口岸核心能力建设通过国家验收，国家进境粮食口岸申报工作扎实推进，港口吞吐能力达到7000万吨。盘锦市与无锡市对口合作框架协议付诸实施，盘营合作确定27个合作事项，与大连国际机场集团合作正式启动。“双招双引”结出丰硕成果，新签约注册项目102个，实际利用外资2.5亿美元、增长25%，实际到位内资220.4亿元，引进各类人才7140人，进出口总额167亿元、增长53.2%。

　　（三）加大力度实施乡村振兴战略，城乡一体化发展走在前列。制定实施乡村振兴战略规划和年度行动计划，现代农业加快发展，新增“蟹稻”标准化养殖面积10万亩、设施农业播种面积7500亩，建设12个亿元以上产业化项目，粮食产量107万吨，实现“十五连丰”；持续加大农村人居环境整治力度，新建维修“四好农村路”196公里，维修改造城乡道路307公里，完成大洼区客运公交一体化改造，新建改造农村厕所7693座。盘山县“加快发展县域经济、助推乡村振兴”的经验做法受到国务院第五次大督查的通报表扬，大洼稻蟹产业园成为全省首个国家现代农业产业园。

　　文明城市建设不断提升。完成城镇开发边界控制线划定，城市“双修”和城市设计试点工作开始启动；东外环路部分通车，新建改造城市公园5座，城乡植树造林470万株；京环集团管理模式引领城乡环境卫生实现根本性变革，城乡综合执法一体化示范项目通过国家住建部验收。

　　（四）坚定不移打好“三大攻坚战”，发展不平衡不充分问题得到有效破解。制定债务风险化解方案，化解到期政府债务115.3亿元、隐性债务223.6亿元；多渠道筹资解决养老金缺口问题，保证按时足额发放；严厉打击非法集资、金融诈骗等违法违规行为，全面审计揭示金社裕农集团历史积累的风险隐患，依法妥善解决企业债务违约、经营止损、运营停滞等问题，为3622名职工补发了拖欠工资，确保140家超市恢复运营。自加压力做好扶贫帮困工作，落实9项帮扶工程，国标线以下建档立卡贫困人口保持动态为零。

　　强力抓好中央生态环保督察、国家海洋督察以及生态环保督察“回头看”、国家“绿盾”行动反馈意见整改落实。铁腕落实三个“十条”，打好“散乱污”企业治理、扬尘整治、秸秆禁烧、燃煤小锅炉淘汰、烟花爆竹燃放管理等组合拳，空气优良天数比例达到78.9%；落实四级河（湖）长制，重点河流水质有所改善；开展农用地和重点工业用地详查，土壤污染管控修复取得积极成效。围绕修复湿地生态，严密启动发布通告、政策解读、鼓励转产、法律援助、主动接访、重拳打击等工作程序，依法依规打好“退养还湿”这场硬仗，震慑了非法经营，弘扬了社会正气。

　　（五）倾情倾力保障和改善民生，老百姓获得感幸福感安全感日益增强。坚持量力而行、尽力而为，财政支出用于民生比重达到75%。10件民生实事全面完成。城镇新增就业2.3万人，城镇登记失业率2.7%保持全省最低。农垦职工养老金单位缴费基数提高50%、发放标准提高8.6%，城镇退休职工养老金发放标准提高5.5%。城乡低保提标10%，盘锦成为全省唯一实现城乡低保一体化的城市。

　　隆重召开庆祝教师节大会表彰先进，严肃查处在职教师违规补课行为并曝光一批失德失范教师，推行中小学教师“县管校聘”改革试点，加强农村薄弱学校标准化建设，盘锦全域实现义务教育均衡发展。启动实施市中心医院薪酬制度改革试点，构建4个医联体和2个医共体促进医疗卫生资源共享，市民跨省市就医实现直接结算。全国“百名画家画盘锦”活动有力弘扬了地域文化，我市成为国家公共文化服务体系示范区。

　　全国居家和社区养老服务改革试点全面启动，20个社区日间照料中心、20个农村互助幸福院实施委托运营。全力解决久拖未决的不动产登记历史遗留问题，已有96个小区5.8万户居民获得产权证书；完成1126个弃管小区改造任务；728户超期未回迁家庭喜迁新居。企业退休职工取暖费人均补贴标准由120元提高到500元。

　　严格落实地方党政领导安全生产责任制，有效遏制重特大安全生产事故发生。国家食品安全示范城市创建积极推进，肉菜流通可追溯体系项目通过国家商务部验收，我市成为东北地区首个国家农产品质量安全市。信访矛盾减存控增三年攻坚计划成效显著，省交办信访存量案件化解83%，排名全省第一。扫黑除恶专项斗争形成高压态势，侦破黑恶势力犯罪案件19起，抓捕涉黑涉恶人员163人。按照上级决策部署做好民族宗教工作。非洲猪瘟疫情有效防控并解除封锁。

　　成功举办第四届“红马赛”、第三届冰凌穿越挑战赛。圆满完成省委、省政府赋予的省“十三运”和省残运会承办任务，得到了全省上下的高度赞誉。盘锦体育健儿创造了参加省运会和省残运会的最佳战绩。

　　（六）凝心聚力开展“重强抓”专项行动，政府治理能力和水平持续提升。按照省政府决策部署，扎实开展“重实干、强执行、抓落实”专项行动，做出“1+4”总体工作安排，累计推进实施224项重点任务。其中，“重强抓”专项行动8个方面178项任务中，承接省里的137项任务96.6%通过省政府节点考核，我市自加压力推出的41项任务基本完成；小分队破难题18项改革任务有6项取得阶段性成果，其余12项正在积极推进；“办事难”问题专项整治20项任务、开发区建设专项整治8项任务顺利完成。专项行动开展以来，我们坚持月调度、季推进，真正攻下了一批项目、解决了一批问题、办成了一批大事。“重强抓”专项行动业已成为全市振兴发展的大载体、狠抓落实的总抓手、转变作风的大平台。

　　坚持依法行政，自觉接受市人大及其常委会法律监督、工作监督和市政协民主监督，建立向市人大常委会报告国有资产管理制度，提请市人大常委会制订审议《物业管理条例（草案）》，117件市人大代表建议、197件市政协委员提案全部办结，满意率均达到100%。完成新一届市政府法律顾问、法律专家库成员聘任工作。加大审计监督力度，市直预算单位项目资金压减了13.5%。落实中央八项规定精神成果进一步巩固，反腐败反“四风”保持强劲态势，一批违法违纪案件得到严肃查处。激励各级干部新时代新担当新作为，形成了聚精会神干事创业的强大合力。

　　积极支持国防和军队现代化建设，国防动员、边海防、人防和民兵预备役工作不断加强，双拥共建取得新成效，军地融合发展呈现新局面。

　　各位代表，这些成绩的取得，归功于改革开放40载伟大实践和光辉历程，归功于党中央国务院的亲切关怀、省委省政府和市委的坚强领导，归功于140多万盘锦人民的团结奋斗。在此，我代表市政府，向全市广大工人、农民、知识分子、各级干部，向驻盘部队指战员、武警官兵、公安干警，向各位人大代表、政协委员，向各民主党派和无党派人士、各人民团体，向辽河油田、华锦集团、辽河石化等中省直驻盘单位，向离退休老同志及所有关心支持盘锦改革发展的各界人士，致以崇高的敬意和衷心的感谢！

　　回顾过去一年的工作，我们也清醒认识到，对标习近平总书记指出的“四个短板”，我们还面临一些突出问题。面对内外部环境变化和高质量发展要求，经济下行压力依然较大；体制机制障碍仍是制约发展的根本性问题，深化改革还有待持续涉险滩、攻堡垒、求突破；转方式、调结构任务繁重紧迫，现代服务业发展相对滞后，创新能力还不够强，新旧动能转换“青黄不接”；开放合作进展不快、步伐不大，经济外向依存度仍然较低；人民群众在就业、教育、医疗、生态环境等方面还有诸多不满意的地方；一些干部主动为市场主体考虑和服务的意识比较欠缺，工作中因循守旧、固步自封，不求有功、但求无过，畏首畏尾、敷衍了事，不计成本、不求实效，等等。我们不能为取得的成绩而沾沾自喜，更不能在实现人民对美好生活的向往上有丝毫懈怠，必须风雨兼程、再接再厉解决好这些前进中的问题。这是时代的要求、人民的期待。

　　二、2019年形势任务

　　今年是新中国成立70周年，是决胜全面建成小康社会第一个百年奋斗目标的关键之年，是盘锦冲刺“十三五”、推进高质量发展、乘势闯关追赶的重要一年。乘着改革开放40年的浩荡东风，面对新时代、新矛盾、新目标、新部署，我们仍然处于不进则退、稍慢也退的阶段，既面临诸多风险挑战，也面临前所未有的发展机遇。只要全市上下坚定信心、保持定力，一起拼博、一起奋斗，就一定能够战胜前进道路上的任何艰难险阻，推动盘锦成为解放思想的排头兵、高质量发展的领跑者。

　　今年政府工作的总体要求是：以习近平新时代中国特色社会主义思想为指导，深入学习贯彻党的十九大精神，全面落实习近平总书记在辽宁考察时和在深入推进东北振兴座谈会上的重要讲话精神，认真贯彻中央经济工作会议精神和省委经济工作会议、市委七届十次全会暨市委经济工作会议精神，统筹推进“五位一体”总体布局和协调推进“四个全面”战略布局，深入推进“一带五基地”建设和“五大区域发展战略”实施，落实“五个坚持”，继续打好“三大攻坚战”，聚焦补齐“四个短板”，牢固树立效率效益、内涵品质、生态保护“三个导向”，重实干、强执行、抓落实，全力推进“1+8+2”全链条部署、“三项牵动性工作”和“六项基础性工作”落地见效，统筹做好稳增长、促改革、调结构、惠民生、防风险工作，进一步稳就业、稳金融、稳外贸、稳外资、稳投资、稳预期，提高人民群众获得感、幸福感、安全感，保持经济持续健康发展和社会大局稳定，为全面建成小康社会收官打下决定性基础，以优异成绩庆祝中华人民共和国成立70周年。

　　今年全市经济社会发展的主要预期目标是：地区生产总值增长7.5％，固定资产投资增长10％，规模以上工业增加值增长8.5%，社会消费品零售总额增长7%，一般公共预算收入增长10%，城乡居民收入增长和经济增长同步。

　　做好今年工作，我们要重点把握好以下六个方面：

　　第一，务必在解放思想上有突破。我们要倍加珍惜、长期坚持改革开放40年积累的“九条宝贵经验”，紧扣市委七届十次全会暨市委经济工作会议确定的“五个原则”，立足“九破九立”，进一步解放思想、转变观念，切实把思想和行动统一到“三个下功夫”“五个有利于”上来，与时俱进地把振兴发展大业和各项工作不断推向前进，努力成为解放思想的排头兵。

　　第二，务必在稳中求进上有定力。我们要紧紧围绕“六个稳”，遵循“五个必须”，保持战略定力，坚决守住经济、政治、文化、社会、生态文明建设底线红线，聚焦“三项牵动性工作”和“六项基础性工作”求进求变求突破，保持经济社会持续向好态势，加快形成营商环境好、创新能力强、生态环境优、发展活力足的全面振兴新局面。

　　第三，务必在高质量发展上有成效。我们要始终坚持质量第一、效益优先，加快构建多点支撑、多业并举、多元发展的产业发展新格局，改造升级“老字号”，深度开发“原字号”，培育壮大“新字号”，改变“四多四少”状况，努力实现绿色发展、节约发展、高效发展，显著增强全市经济质量优势，奋力争当高质量发展的领跑者。

　　第四，务必在供给侧结构性改革上有进展。我们要坚定不移把发展的着力点放在实体经济上，围绕三次产业融合发展和结构优化升级，重点在“巩固、增强、提升、畅通”上下真功用狠劲求实效，切实巩固“三去一降一补”成果，不断增强微观主体活力，着力提升产业链水平，大力畅通经济循环，在凤凰涅槃、腾笼换鸟中行稳致远发展。

　　第五，务必在抓重点补短板强弱项上有作为。我们要坚持问题导向、目标引领，对照上级新部署新要求，对照百姓新需求新期盼，对照先进地区好经验好做法，对照更好发挥老优势争创新优势，发现和解决发展不平衡不充分的突出问题，奋力打好“三大攻坚战”，加快补齐拉长“四个短板”，再创盘锦新的辉煌。

　　第六，务必在实施标准化规范化系列化建设上有提升。我们要大力实施标准化战略，健全产品、工程、营商环境、生态环保、安全生产、社会治理、智慧城市等领域规范，提升就业、教育、医疗、文化、育幼、养老、居住、社保等领域服务水平，推动重点领域率先实现标准化，促进各方面工作走向规范化、克服碎片化、实现系列化。

　　三、2019年工作安排

　　（一）推动经济发展实现新突破。牢牢抓住经济建设这个中心，积极扩大有效投资发挥关键作用，切实扩大消费需求发挥基础作用，不断推动盘锦经济创新发展、转型发展、优质发展。

　　毫不动摇抓项目。坚持产业为先、项目为王，把2019年作为项目建设年，成立重大项目推进组，落实重点项目三级领导定点联系责任制，建立项目管家机制，创新招商方式，大力实施谋划一批、落地一批、开工一批、投产一批的“四个一批”工程。摸清闲置土地、厂房、楼宇、烂尾楼底数，启动于楼地区综合开发，最大限度盘活存量资源。促进华锦阿美项目、宝来巴塞尔项目开工建设，做好中石油液化天然气接收站项目推进工作。全市实施重点建设项目335个，其中投资10亿元以上项目35个、亿元以上项目206个、5000万元以上项目94个。

　　坚定不移调结构。全面落实“链长制”，实施技术改造和智能制造两大工程提升产业链水平，突出抓好浩业化工芳烃、陕汽金玺冷链装备、长春化工锂电池铜箔、中科曙光辽宁基地等10个重点项目，促进石化产业沿着炼化一体化、精细化、绿色化方向形成新的竞争优势，推动装备制造产业在智能化、高端化方面实现新的突破。开展服务业提质行动，做精餐饮、住宿、便民服务3个传统行业，做强电子商务、商贸物流2个现代产业，做大健康美容、教育培训、会展经济等一批新增长点，重点培育特色商业街区8条，打造年交易额超亿元的企业电商交易平台3个。完善促进房地产市场平稳健康发展的长效机制，实现房地产投资、商品房销售额双提升。

　　全域联动促旅游。围绕做大红海滩、唱响芦苇荡，发挥2000万元专项资金的撬动效应，推进旅游资源整合，加快创建国家全域旅游示范区。力推红海滩风景廊道晋升为5A级景区，做精县镇10个重点景区，新增10个A级景区，集中打造30个旅游特色村，打造36条旅游特色线路，开展5个主题文化旅游活动。发展休闲度假游，规划建设鼎翔康养小镇，抓好玉桂园水陆游乐等20个重点项目建设。办好冰凌穿越挑战赛、辽河湿地冰雪节、北旅田园冰雪大世界，促进寒地冰雪旅游加快发展。旅游总人次、总收入同步增长15%以上。

　　（二）聚力改革攻坚激发新活力。坚持向改革要动力，以增强微观主体活力为重点，推动重要领域和关键环节改革走深走实。

　　深化政府机构改革。推进各单位新“三定”方案落实，完成机构改革后续工作。完成县区、镇街机构改革任务。完善综合执法管理体制，推进跨领域跨部门综合执法。

　　深化国资国企改革。推进油地融合、央地合作，支持辽河油田千万吨稳产、华锦集团改革创新、辽河石化环烷基润滑油加氢技术改造项目建设。做好驻盘央企“三供一业”分离移交项目维修改造提升工作，完成鼎翔集团厂办大集体改革后续任务。坚持从管企业向管资本转变，积极推进市属国有企业集团健全公司法人治理结构，开展对外合资合作，统筹推进混合所有制改革、专业化重组和“三项制度”改革。重点市属国有企业新增股权多元化或混合所有制企业8户以上，资产负债率降低2个百分点，“三项费用”压减2个百分点，净资产增长3%以上，营业收入增长8%以上，利润增长10%以上，上缴国有资本收益增长8%以上。

　　加快民营经济发展。全力落实全市加快民营企业发展大会精神，全面实施系列配套政策，着力构建亲清新型政商关系，建立联系帮扶民营企业常态化机制，培养造就高素质盘商队伍。实施个转企、小升规、规升巨培育行动，促进500户个体工商户升级为企业、30户小微企业升级为规上企业、5户规上企业升级为超亿元“小巨人”企业。

　　（三）优化营商环境争创新优势。坚持对标国际一流标准、国内最高水平，更多采取改革的办法，更多运用市场化、法治化手段，全面发力打好重塑环境的攻坚战、持久战。

　　提升政务环境。全面推广复制中国（辽宁）自贸试验区制度创新经验，加大“放管服”改革力度，从根本上解决“办事难”问题，确保政务服务标准化试点通过国家验收。优化审批流程，一般工程建设项目审批时限压减至50个工作日以内；持续推进“一网、一门、一次”改革，市县两级80%以上审批和服务事项实现网上办理，全部事项实现“只进一扇门”办理，其中70%以上事项实现一窗受理集成服务，至少600个高频事项实现“最多跑一次”。推进市县两级政务服务大厅标准统一、业务互通、网络互联，加快实现审批服务跨层级跨区域通办，做到企业和群众办事“就近能办、多点可办、少跑快办”。全面完成“多证合一”“证照分离”等商事制度改革任务。

　　改善金融环境。常态化开展银企对接、产融合作，构建“银政企保”紧密合作的融资担保体系。鼓励有条件的企业发行债券、上市融资，引导各类基金主动对接优秀企业、优质项目。发挥应急转贷资金、风险补偿资金等作用，及时妥善解决企业流动性短缺。推动盘锦银行、农信社增资扩股改革。

　　完善政策环境。落实国家更大规模减税、更明显降费政策，全面清理规范涉企收费，降低企业用电用地用工和物流成本。组织民营企业开展对外经贸合作，与央企集团和市属国企开展项目合作、产品对接。加强政策执行监督，确保出台政策不悬空。

　　规范市场环境。实施净化市场环境专项行动。加强对国有集体资产所有权、经营权和民营企业法人财产权等合法权益保护，完善激励创新的产权制度、知识产权保护制度和促进科技成果转化的体制机制。完善政府守信践诺机制，开展拖欠工程款、未及时供地、优惠政策不兑现等问题专项整治；健全诚信经营激励约束机制，强化企业守信联合激励和失信联合惩戒。

　　（四）促进创新创业增添新动能。坚持创新是第一动力、人才是第一资源，实施创新驱动发展战略，全力争取盘锦高新技术产业开发区晋升为国家级高新区，积极争创国家创新型城市。

　　提升创新能力。围绕推动主导产业高质量发展，健全需求为导向、企业为主体的“产校地企融”一体化创新机制，促进新技术、新组织形式、新产业集群形成和发展，不断增强制造业技术创新能力。深化“市院”战略合作，与中国科学院、大连理工大学、辽宁省农科院、大连海洋大学等科研院所开展全面务实合作，力争在共建开放、协同、高效的共性技术研发平台上实现突破。鼓励引导企业、高校和科研院所开放创新资源、设立众创载体、推动融通创新，提供全方位创新创业服务。加大对中小企业创新支持力度，启动企业自主创新能力提升行动计划，新培育科技型中小企业40家、高新技术企业20家，研发经费投入强度达到2.3%，高新技术产品增加值增长15%以上。全力开展好国家知识产权试点，加强与国家和省知识产权、专利机构对接，引进并转化科技成果60项以上。

　　强化人才支撑。全面落实培养和引进人才的各项政策，在创新创业、安家落户、子女教育、医疗养老等方面创造拴心留人条件，打造“人才金港”，建设人才环境最优市。积极推进盘锦高新区争创全省人才管理改革创新试验区。持续开展“双创盘锦精英荟”“海外学子盘锦行”等招才引智活动，深入落实高校毕业生来盘就业创业优惠政策，吸引更多的优秀人才来盘就业创业。培养造就高素质创业和技术领军人才队伍，培育更多“盘锦工匠”。全年引进各类人才3000人以上。

　　（五）加速乡村振兴构建新格局。坚持以产业兴旺为重点、生态宜居为关键、乡风文明为保障、治理有效为基础、生活富裕为根本，加快推进“五个振兴”，高标准规划建设乡村振兴产业园，促进全域一体化。

　　推进现代农业发展。围绕“产业联盟+基地+农户”模式再造盘锦农业，大力发展订单农业、精品农业、都市型农业。组建龙头企业产业联盟，以大洼国家现代农业产业园、盘山省级农产品加工集聚区2个高标准基地为依托，以大米、河蟹、碱地柿子等5个国家地理标志产品为引领，以鼎翔米业、光合水产等30个龙头企业为牵动，形成精品打市场、市场保订单、龙头结联盟、联盟带基地、基地连农户的农业现代化经营模式。注重培育家庭农场、农民合作社等新型经营主体，开展“互联网+精品农产品”行动、化肥农药减量增效行动，统筹实施良种工程、测土配方施肥工程、水稻全程机械化工程。实施亿元以上农业项目13个，推广高产优质多抗水稻新品种5个以上，化肥农药使用量分别下降5%和10%，设施农业播种面积稳定在3.5万亩以上，新增“蟹稻”标准化养殖面积10万亩，新发展认养农业1万亩，粮食产量稳定在100万吨以上。

　　深化农业农村改革。开展好国家农村集体产权制度改革试点，着力壮大村级集体经济，构建形成带动农民致富奔小康的利益共同体。高标准完成全省农垦改革综合试点任务。推进国有农场居民点住房用地改革试点，进一步盘活农垦建设用地资源。坚持和完善农村承包地“三权分置”制度，抓好农村土地承包经营权证书发放工作。实施农业水价综合改革，促进农民减负和节约用水双赢。

　　打造美丽乡村。扭住村容村貌提升、垃圾污水处理、厕所革命三个重点，深入实施农村人居环境整治三年行动计划，健全美丽乡村建设和管护长效机制。加快城乡基础设施互联互通，完成全域客运公交一体化改造，启动100公里“四好农村路”维修改造项目，建成无害化厕所8000座，建设园林新村50个，力争在城乡垃圾智能化、系统化、配套化分类回收处置上实现突破。筹办好乡村振兴秋季博览会。深入实施“引贤下乡”工程，推动市民下乡、能人回乡、企业兴乡。

　　（六）建设生态文明迈上新台阶。坚持协同推动经济发展和环境保护，强化污染防治和生态建设，力争早日跻身国家生态文明建设示范市行列。

　　实施湿地保护工程。编制湿地保护与生态修复规划纲要，启动实施湿地保护和修复“十项工程”。承办好国际碱蓬草科技研讨大会，联手科研院所、大专院校，综合采取生态补水蓄水、海水调配、碱蓬草种植等措施，全面开展红海滩湿地修复。深化油气开采区治理，加强矿区生态修复。实施引水潮沟清淤，恢复陆海潮汐水道，促进滩涂湿地恢复。强化湿地生物多样性保护，改善湿地生物繁衍栖息生存环境。坚决打赢“退养还湿”战役，推进“引湿入城”，建好小微湿地，做好全国小微湿地工作会议承办工作。

　　实施蓝天工程。继续取缔燃煤小锅炉，实现大洼区、兴隆台区集中高效供热。推广清洁能源使用，散煤替代率达到35%以上。开展柴油货车超标排放专项整治。加强秸秆综合利用，严禁秸秆焚烧。全年空气优良天数比例达到79%。

　　实施碧水工程。坚持河海兼顾、系统治理，促进水环境质量总体提升。全面落实河（湖）长制，启动实施河（湖）治理保护三年行动，加大力度“清四乱”，加快田家、新生2座污水处理厂建设，完成17座镇污水处理厂打包运行，推动村级小型污水处理设施稳定运行，切实整治好绕阳河、清水河、月牙河等8条河流。落实国家和省打好渤海综合治理攻坚战的部署要求，全面清理整治海域污染源，严格管控围填海，有效保护海域生态环境。

　　实施净土工程。全面落实土壤污染防治行动计划，有效防控城市建设用地和农用地土壤环境风险。推进盘山县、辽东湾新区再生资源产业园建设。加强重点监管企业所在区域土壤质量监管，启动北燃老厂区土壤修复。

　　建设宜居城市。启动新一轮国土空间规划编制工作，加快推进多规合一、多评合一、多图联审。加大投入补齐基础设施和公共服务设施建设短板，实施城市基础设施建设4项工程，做好阜盘铁路工程前期工作，支持京哈高速盘锦段扩容改造开工建设，维修改建5条公路，启动盘锦火车站东站区改造。深入开展市容市貌综合整治提升工程，完成背街小巷、老旧小区和弃管小区改造任务，开展“物业服务提升年”活动。建设城市绿道16公里，植树造林130万株。推进智慧城市建设，促进人工智能、工业互联网、物联网等新型基础设施建设不断完善，加快5G商用步伐，完成地下管网管理信息系统建设。积极推进盘山县撤县设区，全力争创国家新型智慧城市示范市、全国人居环境最优市，加快创建国家公交都市示范市，常态化建设全国文明城市,争取蝉联全国双拥模范城。

　　（七）扩大开放合作拓展新空间。坚持适应新形势、把握新特点，深度融入国家重大发展战略，积极参与辽宁“一带一路”综合试验区、中国—中东欧“16+1”经贸合作示范区和“三大通道”建设，全面扩大高水平开放。

　　打造辽东湾开放高地。落实举全市之力发展新区、小分队常态化破难题工作机制，坚持先产业后城市、先二产再三产，实施“港产城校”联动战略，奋力打造形成全市高质量发展的龙头和引擎、适应产业发展的新型城区。依托兵器华锦集团、北燃公司、长春石化等骨干企业，加快建设辽东湾世界级石化及精细化工产业基地。推进京粮物流基地等15个项目开工建设，忠旺铝挤压型材等15个项目竣工投产，力争再引进亿元以上项目20个。实施20项基础设施、公共设施项目。新区规上工业增加值增长12%，固定资产投资增长25%，各项主要经济指标走在全省国家级开发区前列。

　　建设现代化港口。抓住辽宁港口集团组建运营重大机遇，进一步提升盘锦港能力能级，港口吞吐能力达到8000万吨。争取将盘锦港纳入全省战略发展重点港区，落实好拓宽型10万吨级航道新增债券事项确保如期开工建设，加快推进国家进境粮食指定口岸获批，打造国家“北粮南运”重要枢纽。

　　持续引进来走出去。立足国家和省级战略平台，争取更多的政策、项目、资金支持。着力放大开发区、园区“筑巢引凤”效应，完善提升4个省级开发区平台功能，打造招商引资的主战场、高端产业发展的集聚地。深入实施“双招双引”工程，深耕韩日俄、开拓东南亚、聚焦港澳台，紧盯京津冀、长三角、珠三角，实现招商引资的更大突破，全年签约注册项目力争超过100个，其中投资10亿元以上项目5个、5亿元以上项目10个、亿元以上项目50个，实际到位内外资增长10%。积极推进3个省级外贸转型升级基地建设，支持盘锦成济等外贸综合服务企业加快发展，促进外贸出口稳定增长。借助央企国外市场资源，带动我市石油装备等优势产能走出去。

　　扩大区域对接合作。认真贯彻落实省委、省政府关于大力发展飞地经济的部署要求，打破行政区划限制，积极承接市外飞地项目转移，引入飞地项目20个。加强与无锡市在干部人才交流、产业对接互补、合作平台共建等领域对口合作，深化与营口市在基础设施、相关产业、科技创新、社会事业等方面协同合作，积极推进与锦州市、阜新市协同协作，抓好与大连国际机场集团的战略合作，力争在关键领域取得突破性成果。

　　（八）保障改善民生提升新水平。坚持完善制度、守住底线，精心做好各项民生工作，集中办好10件民生实事，让全市人民有更多更直接更实在的获得感、幸福感、安全感。

　　抓教育提素质。加大教育投入力度，促进各级各类教育均衡发展。办好学前教育，新增公办幼儿园学位3800个，开展规范化儿童早期教育服务试点。支持大连理工大学盘锦校区“双一流”建设。推进盘锦职业技术学院“双高”建设，扩建升级盘锦高级技工学校。推广中小学教师“县管校聘”等改革，严肃查处教师失德失范行为，采取政府、学校、家庭协作方式解决义务教育学校课后服务问题。

　　抓创业带就业。实施就业优先政策，突出抓好高校毕业生、农民工、退役军人等重点群体就业。开展标准化规范化医养护理、家政服务等职业技能培训。扩大“一村一技”培训试点范围，扶持创业带头人400人。全年城镇新增就业2.5万人以上，城镇登记失业率控制在3%以内，零就业家庭始终保持动态为零。继续实施城乡居民和各类群体收入增长三年行动计划，确保收入水平稳步提升。

　　抓社保兜底线。推进精准帮扶，完善低保对象认定办法，规范特困人员救助供养制度，提高低保和特困人员救助供养标准，进一步提升精准帮扶质量。开展残疾儿童康复和家庭帮扶等7项工作。推进医养深度融合，抓好全国居家和社区养老服务改革试点，建设5个以上社区养老服务综合体、50个社区老年人日间照料中心、130个农村社区互助幸福院，实现城乡社区养老设施全覆盖。

　　抓卫生促健康。坚持“三医”联动，深化以人事薪酬制度改革为重点的城市公立医院改革，推进医联体、分级诊疗全覆盖，推广家庭医生签约服务。将城镇居民基本医疗保险门诊慢病补助限额增加50%，大病保险起付线降低到1.7万元，统筹解决患者医疗费报销难问题，争取更多救命救急的好药纳入医保。加强传染病疫情防控，大力开展灭蚊等爱国卫生运动。推进国家卫生城镇创建。持续办好“红马赛”，打造徒步大会等群众体育活动品牌，筹办好市第八届运动会。加强食品药品安全治理体系建设，强化食品和药械生产经营质量安全监管，争创国家食品安全示范城市。

　　抓文化强内涵。巩固提升国家公共文化服务体系示范区创建成果，继续深化文化体制机制改革，促进文化馆、图书馆等公共文化设施惠民效能提升。启动市融媒体中心升级改造工程。加强辽河口地域文化三大产业基地建设，发展文化创意、文化会展、苇艺草编、特色民俗等产业，开展四季系列文化消费活动，创建国家文化消费试点市。

　　抓管理防风险。立足坚定、可控、有序、适度，把发展作为化债的根本手段，坚持化债与优债并举，规范政府举债融资机制，化短为长、化高为低，化存量、遏增量，促进资产和资本同步增长。设立债务调节基金，打造辽东湾新区和盘锦高新区两个“AA+”平台。抓住国家较大幅度增加地方政府专项债券规模机遇，务求在相关债券发行上有所作为。牢固树立让百姓过好日子、政府过紧日子的思想，市直部门支出预算再压减10%以上。做好养老保险省和国家统筹相关工作，确保按时足额发放。

　　抓稳定促和谐。进一步做好民族宗教事务、退役军人服务管理。实施回迁难、不动产登记难“两清零”行动，确保1382户动迁家庭得到安置，不动产登记历史遗留问题全部解决。严格犬类等宠物管理。深入开展扫黑除恶专项斗争，加强智慧公安、效率公安、忠诚公安建设，完善社会化治安防控体系、反恐防范体系，争创全国打击盗抢骗提升破案率试点市。落实“五级书记抓信访”责任制，深入实施信访矛盾减存控增三年攻坚计划。严格落实农民工工资“治欠保支”三年行动计划。组建市人民调解中心和各类专业调解委员会，进一步完善公共法律服务体系。严格落实安全生产责任制，开展危化品生产运输储存专项整治，下更大气力抓好生产安全和交通安全。

　　各位代表，盘锦改革发展走进新时代、迎来新机遇，政府工作必须彰显新气象、展现新作为。

　　我们要旗帜鲜明讲政治。牢固树立“四个意识”，坚定“四个自信”，坚决做到“两个维护”，坚决服从、坚决执行、坚决落实党中央、国务院大政方针，上下贯通、左右衔接、目标一致地把省委、省政府和市委决策部署落到实处，确保市政府各项工作始终沿着正确的政治方向前进。

　　我们要牢记宗旨为人民。全面落实以人民为中心的发展思想，永葆为人民谋幸福初心，永做为百姓办好事公仆，直面问题、化解难题，干在当下、利及长远，始终顺应民心、尊重民意、关注民情、致力民生，让老百姓吃得放心、穿得称心、用得舒心、生活安心，让政府一切工作和决策都顺民心、合民意、接地气。

　　我们要依法行政履好职。积极推进法治政府建设，全面正确履行政府职能，依法接受市人大及其常委会法律监督、工作监督，自觉接受市政协的民主监督，主动接受群众监督、舆论监督，推进审计监督全覆盖，让权力在阳光下运行，让服务在阳光下提升。

　　我们要实干高效有作为。“一勤天下无难事”，事业都是干出来的。要不断完善激励政策、容错纠错机制，实施精准问责，引导各级干部自觉践行新时代辽宁精神，天天有激情、时时在状态、处处显担当，恪尽职守、勤勉敬业，勇于斗争、敢于担当，重实干、强执行、抓落实，让全年工作任务一项一项落地见效。

　　我们要廉洁从政守底线。全面履行管党治党主体责任，严格落实“一岗双责”，以永远在路上的坚韧和执着，锲而不舍落实中央八项规定精神，严厉惩治重点领域、关键环节及群众身边的腐败和作风问题，始终保持艰苦奋斗、勤俭节约的优良传统，努力做到干部清正、政府清廉、政治清明。

　　各位代表！实现盘锦全面振兴、全方位振兴，使命更光荣，任务更艰巨，挑战更严峻，工作更伟大。让我们更加紧密团结在以习近平同志为核心的党中央周围，在省委、省政府和市委的坚强领导下，以坚如磐石的信心、只争朝夕的劲头、坚韧不拔的毅力，埋头苦干、攻坚克难，奋力谱写盘锦高质量发展的崭新篇章，以优异成绩迎接新中国成立70周年！

　　2019年1月9日在盘锦市第八届人民代表大会第三次会议上

　　市长	汤方栋

各位代表：

　　现在，我代表盘锦市人民政府向大会报告工作，请予审议，并请市政协委员和其他列席同志提出意见。

　　一、2018年工作回顾

　　刚刚过去的一年，是贯彻落实党的十九大精神和本届政府依法履职的开局之年。在省委、省政府和市委的坚强领导下，我们坚持以习近平新时代中国特色社会主义思想为指导，深入学习贯彻习近平总书记关于东北、辽宁老工业基地振兴的系列重要讲话精神，认真贯彻落实全省“一带五基地”建设和“五大区域发展战略”决策部署，重实干、强执行、抓落实，较好地完成了市八届人大一次会议确定的主要目标任务，盘锦在新时代呈现出新气象新面貌。

　　（一）牢牢把握第一要务，全市经济正步入高质量发展轨道。多措并举确保经济运行在合理区间，预计全市地区生产总值完成1200亿元，增长5.5%，总量全省第五；一般公共预算收入完成135.7亿元，增长14.2%，其中税收收入占比80.3%，总量全省第四；固定资产投资完成457.5亿元，总量保持全省第三；社会消费品零售总额完成417亿元，增长6%；城镇人均可支配收入39030元，增长7%，位居全省第三；农村人均可支配收入17130元，增长7.5%，位居全省第二。

　　国有经济稳固支撑，民营经济活力释放。辽河油田连续33年保持千万吨规模稳产，近四年来实现首次全面盈利；召开加快民营企业发展大会和产融供需对接会，出台含金量高的配套支持政策，新增民营企业1.7万户，民营经济贡献了58.8%的固定资产投资。坚持省市县领导定点联系推进重点项目，备受瞩目的华锦阿美炼化一体化合作项目在沙特签订重要协议、由准备预备阶段进入加速推进阶段，宝来巴塞尔轻烃综合利用项目、中石油液化天然气工程项目进展顺利，浩业化工高分子材料、中蓝电子产业园二期被列入国家东北振兴三年滚动计划项目篮子，全市亿元以上项目新开工71个、续建207个、竣工82个。

　　全面推行“链长制”促进工业主导产业集群化发展，规上工业增加值增长10%。现代服务业快速成长，盘山快递物流园年处理快件7.3亿单，天天好德石化网等3个工业品平台获评全省特色电商平台；召开旅游业发展大会，出台全域旅游实施意见和支持政策，全市接待国内外游客3048万人次，旅游总收入增长15.1%。坚持创新驱动发展，引进建设产业技术研究院5个，培育科技型中小企业140家、高新技术企业40家，科技成果转化落地61项，高新技术产品增加值增长15%。

　　（二）持之以恒深化改革开放，发展动力活力进一步增强。高标准完成市级机构改革、事业单位改革和开发区整治任务，42个工作部门完成集中挂牌、人员转隶、三定方案制定，301家涉改事业单位整合为37家，开发区由9个优化整合为7个，辽河口经济区委托盘山县管理。“放管服”改革取得新进展，“证照分离”和“双随机、一公开”监管全面推开，市县两级审批服务事项70%以上实现一网一门通办，180个高频事项实现“最多跑一次”。驻盘央企“三供一业”资产及管理权分离移交工作全面完成，鼎翔集团厂办大集体改革积极推进，市属国有企业法人户数和管理层级有效压减，与省属国企开展8个合作项目。农垦改革完成4项国家专项试点任务。县镇财政管理体制改革成效显著，33个镇街建立一级国库，镇级财政收入增长58.9%。推进在建项目和闲置土地“两清理”，盘活闲置土地1100亩。

　　一以贯之举全市之力发展辽东湾，统筹做好开发开放、体制机制创新、社会职能划转等工作，新区成为全省推进自贸区协同发展重点园区。盘锦港随着全省港口资源整合迎来发展新机遇，15个5万吨级泊位投入运营，拓宽型10万吨级航道工程即将开工，口岸核心能力建设通过国家验收，国家进境粮食口岸申报工作扎实推进，港口吞吐能力达到7000万吨。盘锦市与无锡市对口合作框架协议付诸实施，盘营合作确定27个合作事项，与大连国际机场集团合作正式启动。“双招双引”结出丰硕成果，新签约注册项目102个，实际利用外资2.5亿美元、增长25%，实际到位内资220.4亿元，引进各类人才7140人，进出口总额167亿元、增长53.2%。

　　（三）加大力度实施乡村振兴战略，城乡一体化发展走在前列。制定实施乡村振兴战略规划和年度行动计划，现代农业加快发展，新增“蟹稻”标准化养殖面积10万亩、设施农业播种面积7500亩，建设12个亿元以上产业化项目，粮食产量107万吨，实现“十五连丰”；持续加大农村人居环境整治力度，新建维修“四好农村路”196公里，维修改造城乡道路307公里，完成大洼区客运公交一体化改造，新建改造农村厕所7693座。盘山县“加快发展县域经济、助推乡村振兴”的经验做法受到国务院第五次大督查的通报表扬，大洼稻蟹产业园成为全省首个国家现代农业产业园。

　　文明城市建设不断提升。完成城镇开发边界控制线划定，城市“双修”和城市设计试点工作开始启动；东外环路部分通车，新建改造城市公园5座，城乡植树造林470万株；京环集团管理模式引领城乡环境卫生实现根本性变革，城乡综合执法一体化示范项目通过国家住建部验收。

　　（四）坚定不移打好“三大攻坚战”，发展不平衡不充分问题得到有效破解。制定债务风险化解方案，化解到期政府债务115.3亿元、隐性债务223.6亿元；多渠道筹资解决养老金缺口问题，保证按时足额发放；严厉打击非法集资、金融诈骗等违法违规行为，全面审计揭示金社裕农集团历史积累的风险隐患，依法妥善解决企业债务违约、经营止损、运营停滞等问题，为3622名职工补发了拖欠工资，确保140家超市恢复运营。自加压力做好扶贫帮困工作，落实9项帮扶工程，国标线以下建档立卡贫困人口保持动态为零。

　　强力抓好中央生态环保督察、国家海洋督察以及生态环保督察“回头看”、国家“绿盾”行动反馈意见整改落实。铁腕落实三个“十条”，打好“散乱污”企业治理、扬尘整治、秸秆禁烧、燃煤小锅炉淘汰、烟花爆竹燃放管理等组合拳，空气优良天数比例达到78.9%；落实四级河（湖）长制，重点河流水质有所改善；开展农用地和重点工业用地详查，土壤污染管控修复取得积极成效。围绕修复湿地生态，严密启动发布通告、政策解读、鼓励转产、法律援助、主动接访、重拳打击等工作程序，依法依规打好“退养还湿”这场硬仗，震慑了非法经营，弘扬了社会正气。

　　（五）倾情倾力保障和改善民生，老百姓获得感幸福感安全感日益增强。坚持量力而行、尽力而为，财政支出用于民生比重达到75%。10件民生实事全面完成。城镇新增就业2.3万人，城镇登记失业率2.7%保持全省最低。农垦职工养老金单位缴费基数提高50%、发放标准提高8.6%，城镇退休职工养老金发放标准提高5.5%。城乡低保提标10%，盘锦成为全省唯一实现城乡低保一体化的城市。

　　隆重召开庆祝教师节大会表彰先进，严肃查处在职教师违规补课行为并曝光一批失德失范教师，推行中小学教师“县管校聘”改革试点，加强农村薄弱学校标准化建设，盘锦全域实现义务教育均衡发展。启动实施市中心医院薪酬制度改革试点，构建4个医联体和2个医共体促进医疗卫生资源共享，市民跨省市就医实现直接结算。全国“百名画家画盘锦”活动有力弘扬了地域文化，我市成为国家公共文化服务体系示范区。

　　全国居家和社区养老服务改革试点全面启动，20个社区日间照料中心、20个农村互助幸福院实施委托运营。全力解决久拖未决的不动产登记历史遗留问题，已有96个小区5.8万户居民获得产权证书；完成1126个弃管小区改造任务；728户超期未回迁家庭喜迁新居。企业退休职工取暖费人均补贴标准由120元提高到500元。

　　严格落实地方党政领导安全生产责任制，有效遏制重特大安全生产事故发生。国家食品安全示范城市创建积极推进，肉菜流通可追溯体系项目通过国家商务部验收，我市成为东北地区首个国家农产品质量安全市。信访矛盾减存控增三年攻坚计划成效显著，省交办信访存量案件化解83%，排名全省第一。扫黑除恶专项斗争形成高压态势，侦破黑恶势力犯罪案件19起，抓捕涉黑涉恶人员163人。按照上级决策部署做好民族宗教工作。非洲猪瘟疫情有效防控并解除封锁。

　　成功举办第四届“红马赛”、第三届冰凌穿越挑战赛。圆满完成省委、省政府赋予的省“十三运”和省残运会承办任务，得到了全省上下的高度赞誉。盘锦体育健儿创造了参加省运会和省残运会的最佳战绩。

　　（六）凝心聚力开展“重强抓”专项行动，政府治理能力和水平持续提升。按照省政府决策部署，扎实开展“重实干、强执行、抓落实”专项行动，做出“1+4”总体工作安排，累计推进实施224项重点任务。其中，“重强抓”专项行动8个方面178项任务中，承接省里的137项任务96.6%通过省政府节点考核，我市自加压力推出的41项任务基本完成；小分队破难题18项改革任务有6项取得阶段性成果，其余12项正在积极推进；“办事难”问题专项整治20项任务、开发区建设专项整治8项任务顺利完成。专项行动开展以来，我们坚持月调度、季推进，真正攻下了一批项目、解决了一批问题、办成了一批大事。“重强抓”专项行动业已成为全市振兴发展的大载体、狠抓落实的总抓手、转变作风的大平台。

　　坚持依法行政，自觉接受市人大及其常委会法律监督、工作监督和市政协民主监督，建立向市人大常委会报告国有资产管理制度，提请市人大常委会制订审议《物业管理条例（草案）》，117件市人大代表建议、197件市政协委员提案全部办结，满意率均达到100%。完成新一届市政府法律顾问、法律专家库成员聘任工作。加大审计监督力度，市直预算单位项目资金压减了13.5%。落实中央八项规定精神成果进一步巩固，反腐败反“四风”保持强劲态势，一批违法违纪案件得到严肃查处。激励各级干部新时代新担当新作为，形成了聚精会神干事创业的强大合力。

　　积极支持国防和军队现代化建设，国防动员、边海防、人防和民兵预备役工作不断加强，双拥共建取得新成效，军地融合发展呈现新局面。

　　各位代表，这些成绩的取得，归功于改革开放40载伟大实践和光辉历程，归功于党中央国务院的亲切关怀、省委省政府和市委的坚强领导，归功于140多万盘锦人民的团结奋斗。在此，我代表市政府，向全市广大工人、农民、知识分子、各级干部，向驻盘部队指战员、武警官兵、公安干警，向各位人大代表、政协委员，向各民主党派和无党派人士、各人民团体，向辽河油田、华锦集团、辽河石化等中省直驻盘单位，向离退休老同志及所有关心支持盘锦改革发展的各界人士，致以崇高的敬意和衷心的感谢！

　　回顾过去一年的工作，我们也清醒认识到，对标习近平总书记指出的“四个短板”，我们还面临一些突出问题。面对内外部环境变化和高质量发展要求，经济下行压力依然较大；体制机制障碍仍是制约发展的根本性问题，深化改革还有待持续涉险滩、攻堡垒、求突破；转方式、调结构任务繁重紧迫，现代服务业发展相对滞后，创新能力还不够强，新旧动能转换“青黄不接”；开放合作进展不快、步伐不大，经济外向依存度仍然较低；人民群众在就业、教育、医疗、生态环境等方面还有诸多不满意的地方；一些干部主动为市场主体考虑和服务的意识比较欠缺，工作中因循守旧、固步自封，不求有功、但求无过，畏首畏尾、敷衍了事，不计成本、不求实效，等等。我们不能为取得的成绩而沾沾自喜，更不能在实现人民对美好生活的向往上有丝毫懈怠，必须风雨兼程、再接再厉解决好这些前进中的问题。这是时代的要求、人民的期待。

　　二、2019年形势任务

　　今年是新中国成立70周年，是决胜全面建成小康社会第一个百年奋斗目标的关键之年，是盘锦冲刺“十三五”、推进高质量发展、乘势闯关追赶的重要一年。乘着改革开放40年的浩荡东风，面对新时代、新矛盾、新目标、新部署，我们仍然处于不进则退、稍慢也退的阶段，既面临诸多风险挑战，也面临前所未有的发展机遇。只要全市上下坚定信心、保持定力，一起拼博、一起奋斗，就一定能够战胜前进道路上的任何艰难险阻，推动盘锦成为解放思想的排头兵、高质量发展的领跑者。

　　今年政府工作的总体要求是：以习近平新时代中国特色社会主义思想为指导，深入学习贯彻党的十九大精神，全面落实习近平总书记在辽宁考察时和在深入推进东北振兴座谈会上的重要讲话精神，认真贯彻中央经济工作会议精神和省委经济工作会议、市委七届十次全会暨市委经济工作会议精神，统筹推进“五位一体”总体布局和协调推进“四个全面”战略布局，深入推进“一带五基地”建设和“五大区域发展战略”实施，落实“五个坚持”，继续打好“三大攻坚战”，聚焦补齐“四个短板”，牢固树立效率效益、内涵品质、生态保护“三个导向”，重实干、强执行、抓落实，全力推进“1+8+2”全链条部署、“三项牵动性工作”和“六项基础性工作”落地见效，统筹做好稳增长、促改革、调结构、惠民生、防风险工作，进一步稳就业、稳金融、稳外贸、稳外资、稳投资、稳预期，提高人民群众获得感、幸福感、安全感，保持经济持续健康发展和社会大局稳定，为全面建成小康社会收官打下决定性基础，以优异成绩庆祝中华人民共和国成立70周年。

　　今年全市经济社会发展的主要预期目标是：地区生产总值增长7.5％，固定资产投资增长10％，规模以上工业增加值增长8.5%，社会消费品零售总额增长7%，一般公共预算收入增长10%，城乡居民收入增长和经济增长同步。

　　做好今年工作，我们要重点把握好以下六个方面：

　　第一，务必在解放思想上有突破。我们要倍加珍惜、长期坚持改革开放40年积累的“九条宝贵经验”，紧扣市委七届十次全会暨市委经济工作会议确定的“五个原则”，立足“九破九立”，进一步解放思想、转变观念，切实把思想和行动统一到“三个下功夫”“五个有利于”上来，与时俱进地把振兴发展大业和各项工作不断推向前进，努力成为解放思想的排头兵。

　　第二，务必在稳中求进上有定力。我们要紧紧围绕“六个稳”，遵循“五个必须”，保持战略定力，坚决守住经济、政治、文化、社会、生态文明建设底线红线，聚焦“三项牵动性工作”和“六项基础性工作”求进求变求突破，保持经济社会持续向好态势，加快形成营商环境好、创新能力强、生态环境优、发展活力足的全面振兴新局面。

　　第三，务必在高质量发展上有成效。我们要始终坚持质量第一、效益优先，加快构建多点支撑、多业并举、多元发展的产业发展新格局，改造升级“老字号”，深度开发“原字号”，培育壮大“新字号”，改变“四多四少”状况，努力实现绿色发展、节约发展、高效发展，显著增强全市经济质量优势，奋力争当高质量发展的领跑者。

　　第四，务必在供给侧结构性改革上有进展。我们要坚定不移把发展的着力点放在实体经济上，围绕三次产业融合发展和结构优化升级，重点在“巩固、增强、提升、畅通”上下真功用狠劲求实效，切实巩固“三去一降一补”成果，不断增强微观主体活力，着力提升产业链水平，大力畅通经济循环，在凤凰涅槃、腾笼换鸟中行稳致远发展。

　　第五，务必在抓重点补短板强弱项上有作为。我们要坚持问题导向、目标引领，对照上级新部署新要求，对照百姓新需求新期盼，对照先进地区好经验好做法，对照更好发挥老优势争创新优势，发现和解决发展不平衡不充分的突出问题，奋力打好“三大攻坚战”，加快补齐拉长“四个短板”，再创盘锦新的辉煌。

　　第六，务必在实施标准化规范化系列化建设上有提升。我们要大力实施标准化战略，健全产品、工程、营商环境、生态环保、安全生产、社会治理、智慧城市等领域规范，提升就业、教育、医疗、文化、育幼、养老、居住、社保等领域服务水平，推动重点领域率先实现标准化，促进各方面工作走向规范化、克服碎片化、实现系列化。

　　三、2019年工作安排

　　（一）推动经济发展实现新突破。牢牢抓住经济建设这个中心，积极扩大有效投资发挥关键作用，切实扩大消费需求发挥基础作用，不断推动盘锦经济创新发展、转型发展、优质发展。

　　毫不动摇抓项目。坚持产业为先、项目为王，把2019年作为项目建设年，成立重大项目推进组，落实重点项目三级领导定点联系责任制，建立项目管家机制，创新招商方式，大力实施谋划一批、落地一批、开工一批、投产一批的“四个一批”工程。摸清闲置土地、厂房、楼宇、烂尾楼底数，启动于楼地区综合开发，最大限度盘活存量资源。促进华锦阿美项目、宝来巴塞尔项目开工建设，做好中石油液化天然气接收站项目推进工作。全市实施重点建设项目335个，其中投资10亿元以上项目35个、亿元以上项目206个、5000万元以上项目94个。

　　坚定不移调结构。全面落实“链长制”，实施技术改造和智能制造两大工程提升产业链水平，突出抓好浩业化工芳烃、陕汽金玺冷链装备、长春化工锂电池铜箔、中科曙光辽宁基地等10个重点项目，促进石化产业沿着炼化一体化、精细化、绿色化方向形成新的竞争优势，推动装备制造产业在智能化、高端化方面实现新的突破。开展服务业提质行动，做精餐饮、住宿、便民服务3个传统行业，做强电子商务、商贸物流2个现代产业，做大健康美容、教育培训、会展经济等一批新增长点，重点培育特色商业街区8条，打造年交易额超亿元的企业电商交易平台3个。完善促进房地产市场平稳健康发展的长效机制，实现房地产投资、商品房销售额双提升。

　　全域联动促旅游。围绕做大红海滩、唱响芦苇荡，发挥2000万元专项资金的撬动效应，推进旅游资源整合，加快创建国家全域旅游示范区。力推红海滩风景廊道晋升为5A级景区，做精县镇10个重点景区，新增10个A级景区，集中打造30个旅游特色村，打造36条旅游特色线路，开展5个主题文化旅游活动。发展休闲度假游，规划建设鼎翔康养小镇，抓好玉桂园水陆游乐等20个重点项目建设。办好冰凌穿越挑战赛、辽河湿地冰雪节、北旅田园冰雪大世界，促进寒地冰雪旅游加快发展。旅游总人次、总收入同步增长15%以上。

　　（二）聚力改革攻坚激发新活力。坚持向改革要动力，以增强微观主体活力为重点，推动重要领域和关键环节改革走深走实。

　　深化政府机构改革。推进各单位新“三定”方案落实，完成机构改革后续工作。完成县区、镇街机构改革任务。完善综合执法管理体制，推进跨领域跨部门综合执法。

　　深化国资国企改革。推进油地融合、央地合作，支持辽河油田千万吨稳产、华锦集团改革创新、辽河石化环烷基润滑油加氢技术改造项目建设。做好驻盘央企“三供一业”分离移交项目维修改造提升工作，完成鼎翔集团厂办大集体改革后续任务。坚持从管企业向管资本转变，积极推进市属国有企业集团健全公司法人治理结构，开展对外合资合作，统筹推进混合所有制改革、专业化重组和“三项制度”改革。重点市属国有企业新增股权多元化或混合所有制企业8户以上，资产负债率降低2个百分点，“三项费用”压减2个百分点，净资产增长3%以上，营业收入增长8%以上，利润增长10%以上，上缴国有资本收益增长8%以上。

　　加快民营经济发展。全力落实全市加快民营企业发展大会精神，全面实施系列配套政策，着力构建亲清新型政商关系，建立联系帮扶民营企业常态化机制，培养造就高素质盘商队伍。实施个转企、小升规、规升巨培育行动，促进500户个体工商户升级为企业、30户小微企业升级为规上企业、5户规上企业升级为超亿元“小巨人”企业。

　　（三）优化营商环境争创新优势。坚持对标国际一流标准、国内最高水平，更多采取改革的办法，更多运用市场化、法治化手段，全面发力打好重塑环境的攻坚战、持久战。

　　提升政务环境。全面推广复制中国（辽宁）自贸试验区制度创新经验，加大“放管服”改革力度，从根本上解决“办事难”问题，确保政务服务标准化试点通过国家验收。优化审批流程，一般工程建设项目审批时限压减至50个工作日以内；持续推进“一网、一门、一次”改革，市县两级80%以上审批和服务事项实现网上办理，全部事项实现“只进一扇门”办理，其中70%以上事项实现一窗受理集成服务，至少600个高频事项实现“最多跑一次”。推进市县两级政务服务大厅标准统一、业务互通、网络互联，加快实现审批服务跨层级跨区域通办，做到企业和群众办事“就近能办、多点可办、少跑快办”。全面完成“多证合一”“证照分离”等商事制度改革任务。

　　改善金融环境。常态化开展银企对接、产融合作，构建“银政企保”紧密合作的融资担保体系。鼓励有条件的企业发行债券、上市融资，引导各类基金主动对接优秀企业、优质项目。发挥应急转贷资金、风险补偿资金等作用，及时妥善解决企业流动性短缺。推动盘锦银行、农信社增资扩股改革。

　　完善政策环境。落实国家更大规模减税、更明显降费政策，全面清理规范涉企收费，降低企业用电用地用工和物流成本。组织民营企业开展对外经贸合作，与央企集团和市属国企开展项目合作、产品对接。加强政策执行监督，确保出台政策不悬空。

　　规范市场环境。实施净化市场环境专项行动。加强对国有集体资产所有权、经营权和民营企业法人财产权等合法权益保护，完善激励创新的产权制度、知识产权保护制度和促进科技成果转化的体制机制。完善政府守信践诺机制，开展拖欠工程款、未及时供地、优惠政策不兑现等问题专项整治；健全诚信经营激励约束机制，强化企业守信联合激励和失信联合惩戒。

　　（四）促进创新创业增添新动能。坚持创新是第一动力、人才是第一资源，实施创新驱动发展战略，全力争取盘锦高新技术产业开发区晋升为国家级高新区，积极争创国家创新型城市。

　　提升创新能力。围绕推动主导产业高质量发展，健全需求为导向、企业为主体的“产校地企融”一体化创新机制，促进新技术、新组织形式、新产业集群形成和发展，不断增强制造业技术创新能力。深化“市院”战略合作，与中国科学院、大连理工大学、辽宁省农科院、大连海洋大学等科研院所开展全面务实合作，力争在共建开放、协同、高效的共性技术研发平台上实现突破。鼓励引导企业、高校和科研院所开放创新资源、设立众创载体、推动融通创新，提供全方位创新创业服务。加大对中小企业创新支持力度，启动企业自主创新能力提升行动计划，新培育科技型中小企业40家、高新技术企业20家，研发经费投入强度达到2.3%，高新技术产品增加值增长15%以上。全力开展好国家知识产权试点，加强与国家和省知识产权、专利机构对接，引进并转化科技成果60项以上。

　　强化人才支撑。全面落实培养和引进人才的各项政策，在创新创业、安家落户、子女教育、医疗养老等方面创造拴心留人条件，打造“人才金港”，建设人才环境最优市。积极推进盘锦高新区争创全省人才管理改革创新试验区。持续开展“双创盘锦精英荟”“海外学子盘锦行”等招才引智活动，深入落实高校毕业生来盘就业创业优惠政策，吸引更多的优秀人才来盘就业创业。培养造就高素质创业和技术领军人才队伍，培育更多“盘锦工匠”。全年引进各类人才3000人以上。

　　（五）加速乡村振兴构建新格局。坚持以产业兴旺为重点、生态宜居为关键、乡风文明为保障、治理有效为基础、生活富裕为根本，加快推进“五个振兴”，高标准规划建设乡村振兴产业园，促进全域一体化。

　　推进现代农业发展。围绕“产业联盟+基地+农户”模式再造盘锦农业，大力发展订单农业、精品农业、都市型农业。组建龙头企业产业联盟，以大洼国家现代农业产业园、盘山省级农产品加工集聚区2个高标准基地为依托，以大米、河蟹、碱地柿子等5个国家地理标志产品为引领，以鼎翔米业、光合水产等30个龙头企业为牵动，形成精品打市场、市场保订单、龙头结联盟、联盟带基地、基地连农户的农业现代化经营模式。注重培育家庭农场、农民合作社等新型经营主体，开展“互联网+精品农产品”行动、化肥农药减量增效行动，统筹实施良种工程、测土配方施肥工程、水稻全程机械化工程。实施亿元以上农业项目13个，推广高产优质多抗水稻新品种5个以上，化肥农药使用量分别下降5%和10%，设施农业播种面积稳定在3.5万亩以上，新增“蟹稻”标准化养殖面积10万亩，新发展认养农业1万亩，粮食产量稳定在100万吨以上。

　　深化农业农村改革。开展好国家农村集体产权制度改革试点，着力壮大村级集体经济，构建形成带动农民致富奔小康的利益共同体。高标准完成全省农垦改革综合试点任务。推进国有农场居民点住房用地改革试点，进一步盘活农垦建设用地资源。坚持和完善农村承包地“三权分置”制度，抓好农村土地承包经营权证书发放工作。实施农业水价综合改革，促进农民减负和节约用水双赢。

　　打造美丽乡村。扭住村容村貌提升、垃圾污水处理、厕所革命三个重点，深入实施农村人居环境整治三年行动计划，健全美丽乡村建设和管护长效机制。加快城乡基础设施互联互通，完成全域客运公交一体化改造，启动100公里“四好农村路”维修改造项目，建成无害化厕所8000座，建设园林新村50个，力争在城乡垃圾智能化、系统化、配套化分类回收处置上实现突破。筹办好乡村振兴秋季博览会。深入实施“引贤下乡”工程，推动市民下乡、能人回乡、企业兴乡。

　　（六）建设生态文明迈上新台阶。坚持协同推动经济发展和环境保护，强化污染防治和生态建设，力争早日跻身国家生态文明建设示范市行列。

　　实施湿地保护工程。编制湿地保护与生态修复规划纲要，启动实施湿地保护和修复“十项工程”。承办好国际碱蓬草科技研讨大会，联手科研院所、大专院校，综合采取生态补水蓄水、海水调配、碱蓬草种植等措施，全面开展红海滩湿地修复。深化油气开采区治理，加强矿区生态修复。实施引水潮沟清淤，恢复陆海潮汐水道，促进滩涂湿地恢复。强化湿地生物多样性保护，改善湿地生物繁衍栖息生存环境。坚决打赢“退养还湿”战役，推进“引湿入城”，建好小微湿地，做好全国小微湿地工作会议承办工作。

　　实施蓝天工程。继续取缔燃煤小锅炉，实现大洼区、兴隆台区集中高效供热。推广清洁能源使用，散煤替代率达到35%以上。开展柴油货车超标排放专项整治。加强秸秆综合利用，严禁秸秆焚烧。全年空气优良天数比例达到79%。

　　实施碧水工程。坚持河海兼顾、系统治理，促进水环境质量总体提升。全面落实河（湖）长制，启动实施河（湖）治理保护三年行动，加大力度“清四乱”，加快田家、新生2座污水处理厂建设，完成17座镇污水处理厂打包运行，推动村级小型污水处理设施稳定运行，切实整治好绕阳河、清水河、月牙河等8条河流。落实国家和省打好渤海综合治理攻坚战的部署要求，全面清理整治海域污染源，严格管控围填海，有效保护海域生态环境。

　　实施净土工程。全面落实土壤污染防治行动计划，有效防控城市建设用地和农用地土壤环境风险。推进盘山县、辽东湾新区再生资源产业园建设。加强重点监管企业所在区域土壤质量监管，启动北燃老厂区土壤修复。

　　建设宜居城市。启动新一轮国土空间规划编制工作，加快推进多规合一、多评合一、多图联审。加大投入补齐基础设施和公共服务设施建设短板，实施城市基础设施建设4项工程，做好阜盘铁路工程前期工作，支持京哈高速盘锦段扩容改造开工建设，维修改建5条公路，启动盘锦火车站东站区改造。深入开展市容市貌综合整治提升工程，完成背街小巷、老旧小区和弃管小区改造任务，开展“物业服务提升年”活动。建设城市绿道16公里，植树造林130万株。推进智慧城市建设，促进人工智能、工业互联网、物联网等新型基础设施建设不断完善，加快5G商用步伐，完成地下管网管理信息系统建设。积极推进盘山县撤县设区，全力争创国家新型智慧城市示范市、全国人居环境最优市，加快创建国家公交都市示范市，常态化建设全国文明城市,争取蝉联全国双拥模范城。

　　（七）扩大开放合作拓展新空间。坚持适应新形势、把握新特点，深度融入国家重大发展战略，积极参与辽宁“一带一路”综合试验区、中国—中东欧“16+1”经贸合作示范区和“三大通道”建设，全面扩大高水平开放。

　　打造辽东湾开放高地。落实举全市之力发展新区、小分队常态化破难题工作机制，坚持先产业后城市、先二产再三产，实施“港产城校”联动战略，奋力打造形成全市高质量发展的龙头和引擎、适应产业发展的新型城区。依托兵器华锦集团、北燃公司、长春石化等骨干企业，加快建设辽东湾世界级石化及精细化工产业基地。推进京粮物流基地等15个项目开工建设，忠旺铝挤压型材等15个项目竣工投产，力争再引进亿元以上项目20个。实施20项基础设施、公共设施项目。新区规上工业增加值增长12%，固定资产投资增长25%，各项主要经济指标走在全省国家级开发区前列。

　　建设现代化港口。抓住辽宁港口集团组建运营重大机遇，进一步提升盘锦港能力能级，港口吞吐能力达到8000万吨。争取将盘锦港纳入全省战略发展重点港区，落实好拓宽型10万吨级航道新增债券事项确保如期开工建设，加快推进国家进境粮食指定口岸获批，打造国家“北粮南运”重要枢纽。

　　持续引进来走出去。立足国家和省级战略平台，争取更多的政策、项目、资金支持。着力放大开发区、园区“筑巢引凤”效应，完善提升4个省级开发区平台功能，打造招商引资的主战场、高端产业发展的集聚地。深入实施“双招双引”工程，深耕韩日俄、开拓东南亚、聚焦港澳台，紧盯京津冀、长三角、珠三角，实现招商引资的更大突破，全年签约注册项目力争超过100个，其中投资10亿元以上项目5个、5亿元以上项目10个、亿元以上项目50个，实际到位内外资增长10%。积极推进3个省级外贸转型升级基地建设，支持盘锦成济等外贸综合服务企业加快发展，促进外贸出口稳定增长。借助央企国外市场资源，带动我市石油装备等优势产能走出去。

　　扩大区域对接合作。认真贯彻落实省委、省政府关于大力发展飞地经济的部署要求，打破行政区划限制，积极承接市外飞地项目转移，引入飞地项目20个。加强与无锡市在干部人才交流、产业对接互补、合作平台共建等领域对口合作，深化与营口市在基础设施、相关产业、科技创新、社会事业等方面协同合作，积极推进与锦州市、阜新市协同协作，抓好与大连国际机场集团的战略合作，力争在关键领域取得突破性成果。

　　（八）保障改善民生提升新水平。坚持完善制度、守住底线，精心做好各项民生工作，集中办好10件民生实事，让全市人民有更多更直接更实在的获得感、幸福感、安全感。

　　抓教育提素质。加大教育投入力度，促进各级各类教育均衡发展。办好学前教育，新增公办幼儿园学位3800个，开展规范化儿童早期教育服务试点。支持大连理工大学盘锦校区“双一流”建设。推进盘锦职业技术学院“双高”建设，扩建升级盘锦高级技工学校。推广中小学教师“县管校聘”等改革，严肃查处教师失德失范行为，采取政府、学校、家庭协作方式解决义务教育学校课后服务问题。

　　抓创业带就业。实施就业优先政策，突出抓好高校毕业生、农民工、退役军人等重点群体就业。开展标准化规范化医养护理、家政服务等职业技能培训。扩大“一村一技”培训试点范围，扶持创业带头人400人。全年城镇新增就业2.5万人以上，城镇登记失业率控制在3%以内，零就业家庭始终保持动态为零。继续实施城乡居民和各类群体收入增长三年行动计划，确保收入水平稳步提升。

　　抓社保兜底线。推进精准帮扶，完善低保对象认定办法，规范特困人员救助供养制度，提高低保和特困人员救助供养标准，进一步提升精准帮扶质量。开展残疾儿童康复和家庭帮扶等7项工作。推进医养深度融合，抓好全国居家和社区养老服务改革试点，建设5个以上社区养老服务综合体、50个社区老年人日间照料中心、130个农村社区互助幸福院，实现城乡社区养老设施全覆盖。

　　抓卫生促健康。坚持“三医”联动，深化以人事薪酬制度改革为重点的城市公立医院改革，推进医联体、分级诊疗全覆盖，推广家庭医生签约服务。将城镇居民基本医疗保险门诊慢病补助限额增加50%，大病保险起付线降低到1.7万元，统筹解决患者医疗费报销难问题，争取更多救命救急的好药纳入医保。加强传染病疫情防控，大力开展灭蚊等爱国卫生运动。推进国家卫生城镇创建。持续办好“红马赛”，打造徒步大会等群众体育活动品牌，筹办好市第八届运动会。加强食品药品安全治理体系建设，强化食品和药械生产经营质量安全监管，争创国家食品安全示范城市。

　　抓文化强内涵。巩固提升国家公共文化服务体系示范区创建成果，继续深化文化体制机制改革，促进文化馆、图书馆等公共文化设施惠民效能提升。启动市融媒体中心升级改造工程。加强辽河口地域文化三大产业基地建设，发展文化创意、文化会展、苇艺草编、特色民俗等产业，开展四季系列文化消费活动，创建国家文化消费试点市。

　　抓管理防风险。立足坚定、可控、有序、适度，把发展作为化债的根本手段，坚持化债与优债并举，规范政府举债融资机制，化短为长、化高为低，化存量、遏增量，促进资产和资本同步增长。设立债务调节基金，打造辽东湾新区和盘锦高新区两个“AA+”平台。抓住国家较大幅度增加地方政府专项债券规模机遇，务求在相关债券发行上有所作为。牢固树立让百姓过好日子、政府过紧日子的思想，市直部门支出预算再压减10%以上。做好养老保险省和国家统筹相关工作，确保按时足额发放。

　　抓稳定促和谐。进一步做好民族宗教事务、退役军人服务管理。实施回迁难、不动产登记难“两清零”行动，确保1382户动迁家庭得到安置，不动产登记历史遗留问题全部解决。严格犬类等宠物管理。深入开展扫黑除恶专项斗争，加强智慧公安、效率公安、忠诚公安建设，完善社会化治安防控体系、反恐防范体系，争创全国打击盗抢骗提升破案率试点市。落实“五级书记抓信访”责任制，深入实施信访矛盾减存控增三年攻坚计划。严格落实农民工工资“治欠保支”三年行动计划。组建市人民调解中心和各类专业调解委员会，进一步完善公共法律服务体系。严格落实安全生产责任制，开展危化品生产运输储存专项整治，下更大气力抓好生产安全和交通安全。

　　各位代表，盘锦改革发展走进新时代、迎来新机遇，政府工作必须彰显新气象、展现新作为。

　　我们要旗帜鲜明讲政治。牢固树立“四个意识”，坚定“四个自信”，坚决做到“两个维护”，坚决服从、坚决执行、坚决落实党中央、国务院大政方针，上下贯通、左右衔接、目标一致地把省委、省政府和市委决策部署落到实处，确保市政府各项工作始终沿着正确的政治方向前进。

　　我们要牢记宗旨为人民。全面落实以人民为中心的发展思想，永葆为人民谋幸福初心，永做为百姓办好事公仆，直面问题、化解难题，干在当下、利及长远，始终顺应民心、尊重民意、关注民情、致力民生，让老百姓吃得放心、穿得称心、用得舒心、生活安心，让政府一切工作和决策都顺民心、合民意、接地气。

　　我们要依法行政履好职。积极推进法治政府建设，全面正确履行政府职能，依法接受市人大及其常委会法律监督、工作监督，自觉接受市政协的民主监督，主动接受群众监督、舆论监督，推进审计监督全覆盖，让权力在阳光下运行，让服务在阳光下提升。

　　我们要实干高效有作为。“一勤天下无难事”，事业都是干出来的。要不断完善激励政策、容错纠错机制，实施精准问责，引导各级干部自觉践行新时代辽宁精神，天天有激情、时时在状态、处处显担当，恪尽职守、勤勉敬业，勇于斗争、敢于担当，重实干、强执行、抓落实，让全年工作任务一项一项落地见效。

　　我们要廉洁从政守底线。全面履行管党治党主体责任，严格落实“一岗双责”，以永远在路上的坚韧和执着，锲而不舍落实中央八项规定精神，严厉惩治重点领域、关键环节及群众身边的腐败和作风问题，始终保持艰苦奋斗、勤俭节约的优良传统，努力做到干部清正、政府清廉、政治清明。

　　各位代表！实现盘锦全面振兴、全方位振兴，使命更光荣，任务更艰巨，挑战更严峻，工作更伟大。让我们更加紧密团结在以习近平同志为核心的党中央周围，在省委、省政府和市委的坚强领导下，以坚如磐石的信心、只争朝夕的劲头、坚韧不拔的毅力，埋头苦干、攻坚克难，奋力谱写盘锦高质量发展的崭新篇章，以优异成绩迎接新中国成立70周年！
